# Supplementary material for: Are carbon emissions trading and green financial instruments synergistic? -Comprehensive quantitative research based on content analysis
Source: PLoS One. 2024 Mar 7;19(3):e0298601. doi: 10.1371/journal.pone.0298601 (PMC10919601; doi:10.1371/journal.pone.0298601)
Supplement: S1 Data — (ZIP) [file pone.0298601.s001.zip › Data/地级市Z_F工作报告.zip!/╔┬╬≈/╬≈░▓╩╨/└┤╫╘╣1⁄2╓┌║┼í╛┬φ┐╦╩2╛▌═°í┐_2016.docx]

各位代表：

现在，我代表市人民政府向大会报告政府工作，请予审议，并请市政协委员和其他列席人员提出意见。

一、去年和“十二五”工作回顾

过去一年，是西安“十二五”规划的最后一年，也是西安发展历程中极不平凡的一年。面对宏观经济形势异常严峻的不利局面，我们在省委、省政府和市委的坚强领导下，深入学习贯彻党的十八大和十八届三中、四中、五中全会精神，以习近平总书记来陕视察时提出的“追赶超越”和“五个扎实”要求为引领，迎难而上、主动作为，确保经济平稳运行在合理区间，全年实现生产总值5810.03亿元，增长8.2%，一般公共预算收入650.91亿元，增长16.3%，基本完成了市十五届人大五次会议确定的目标任务，实现了“十二五”圆满收官。

回顾“十二五”，我们紧紧围绕建设具有历史文化特色的国际化大都市的奋斗目标，秉持“一张蓝图干到底、两块短板求突破、多措并举惠民生、创新管理促和谐”的理念，扎实推进“五项重点工作”，经济社会发展取得了显著成就。特别是我们举全市之力，干成了事关西安长远发展的“十件大事”——“创文”成功，三星落户，渭北工业区建设全面启动，西安进入地铁时代，成功举办世园会，建成西部首个内陆港，在现行标准下全部贫困人口实现脱贫，丝路联合申遗取得成功，南门地区综合改造圆满完成，国家全面创新改革试验区和自主创新示范区正式获批。城市的承载力、影响力、美誉度和人民的幸福感进一步提升。

——经济综合实力实现新跨越。生产总值连续跨越4000亿元、5000亿元，年均增长10.9%，增速在15个副省级城市中稳居“第一方阵”。人均GDP突破1万美元，预计达到10778美元；财政总收入突破1000亿元，达到1114.98亿元；非公经济占比突破50%，达到52.8%；规模以上工业增加值突破1000亿元，达到1174.67亿元；金融业增加值643.88亿元，占GDP比重突破10%，达到11.1%。12项经济指标较2010年实现“翻番”。

——产业结构调整迈出新步伐。三次产业比由2010年的4.0∶41.9∶54.1调整为3.8∶37.3∶58.9，提前2年完成“十二五”淘汰落后产能任务，单位GDP能耗累计下降18.3%。粮食生产实现“十二连丰”，“一区三带七板块”的都市型现代农业发展格局初步形成。渭北工业区建设全面推进，全市规模以上工业企业数量达到1098户，较2011年增加242户。8个国家级服务业综合改革试点聚集区加快建设，服务业占GDP比重较2010年提高4.8个百分点。战略性新兴产业势头强劲，年均增速预计达到16%左右，高出GDP增速5.1个百分点。半导体产业、生产性服务业、电子商务、物流快递等持续高速增长，“五区一港两基地”的板块带动作用不断增强，新产业、新业态、新模式、新板块正加快成为支撑和带动我市经济转型升级的新引擎。

——美丽西安建设取得新成效。秦岭保护步入法治化轨道，违规采矿、违法建设等破坏生态环境的问题得到有效遏制，景观提升、绿道建设等9项重点工程顺利完成。创建国家森林城市全面达标，共计造林36.48万亩。新建公园30个、绿地广场363个，新增城市绿地1931万平方米，建成区绿化覆盖率从37%提高到42.5%，人均公园绿地面积从9.5平方米提高到11.6平方米。“八水润西安”工程扎实推进，渭河西安段“三年变清”目标如期完成，氵皂河污染治理实现达标，新增生态水面9573亩、湿地面积2.1万亩，新建、改扩建污水处理厂13座，城市污水处理率达到95.5%。建成国家级生态区2个、省级以上生态示范区县3个、市级以上生态镇村209个。特别是，2013年初我市在全国率先提出并实施了治污减霾工作，将其列为环境治理和民生改善的头号工程，累计投入各级财政资金75.6亿元、拆改燃煤锅炉1335台、淘汰黄标车及老旧车15万辆，空气质量持续改善，优良天数从2013年的138天增加至去年的251天，“西安蓝”逐渐成为市民生活的新常态。

——城市综合承载力得到新提升。大力推进主城区“南优、北拓、东延、西联”战略，深入实施古城复兴计划、缓堵保畅工程和城市精细化管理，全市建成区面积从395平方公里增加到522平方公里，城镇化率从69%提高到73%左右，累计完成城建投资1531.4亿元，是“十一五”的1.7倍。南门区域综合改造被评为全国历史文化名城建设重大示范工程，汉长安城未央宫遗址等5处历史古迹入选世界文化遗产。地铁1、2号线建成运营，3号线实现长轨贯通，通车里程达到52公里，日均客运量接近100万人次。改造城市道路170余条，新建人行天桥、地下通道41座，人均城市道路面积达到18.1平方米，较2010年提高2.5平方米。投放公共自行车4.2万辆，服务网点实现主城区全覆盖。新建高速公路3条，改造国省道干线公路270公里，建成等级客运场站5个，农村二级公路实现全覆盖，全市公路通车总里程达到1.3万公里。铁路北客站建成投用，西安成为国内重要的高铁枢纽。启动实施10项水源工程，李家河水源工程建成供水，新增城市日供水能力30万吨。建成750千伏西安南输变电工程，累计新增110千伏变电容量364.1万千伏安，新增年供气能力6亿立方米，新增集中供热面积1.07亿平方米，分别是“十一五”的1.2、1.4和3.2倍。9个省市级重点示范镇和6个文化旅游名镇加快建设，提前完成我市《建设社会主义新农村行动纲要》确定的目标任务。特别是经过18年不懈努力，我市成功摘得“全国文明城市”桂冠，这是870万西安人民的光荣和骄傲。

——全面深化改革释放新动能。坚持下好简政放权“先手棋”，制定公布市级行政许可目录，分四批取消、下放、调整了312项行政事权。扎实推进“营改增”扩围试点和工商登记制度改革，新增企业数量呈现“井喷”之势，各类市场主体总数达到63.8万户，较2010年增加29.7万户。启动新一轮国资国企改革，混合所有制试点有序推进，西纺集团与五环集团完成重组整合。城市网格化管理全面推开，建立了“两级政府、三级管理、四级网络”的大城管格局。农村综合改革有序推进，土地确权登记工作基本完成，流转土地56.3万亩。持续加大统筹科技资源改革力度，累计实现技术成果交易额2120亿元，科技企业小巨人总数超过700家，高新技术产业增加值占GDP比重达到14.5%，较2010年提高3.3个百分点，去年专利申请量突破6万件，是2010年的4.2倍。此外，政府机构改革、公车改革、区县公立医院改革、大学区管理制改革、司法改革等关键领域改革也取得了明显成效。

——对外开放有了新突破。丝绸之路经济带新起点建设全面启动。欧亚经济论坛被确定为国家“一带一路”建设十大平台之一。成功举办三届丝绸之路经济带城市圆桌会，倡导发布《西安宣言》。“长安号”实现常态化运营。“西安港”成为我国首个拥有国际国内“双代码”的内陆港，并正式获批国家一类陆路开放口岸。西安综合保税区、高新综合保税区封关运行。西安咸阳国际机场二期扩建顺利完成，开通国际航线36条，成为西北首个72小时过境免签城市。浐灞生态区获批建设领事馆区，韩国、泰国、柬埔寨分别设立驻西安总领事馆，国际友好城市增至27个。累计举办规模以上会展活动836个，展会成交额达到5768.4亿元。去年全市实际利用外资突破40亿美元，进出口总值252.6亿美元，服务外包合同额14.9亿美元，分别是2010年的2.6、3.5和5.1倍。我市荣登联合国“全球最具发展潜力新兴城市”榜单。

——民生改善增进新福祉。全市财政支出和新增财力的80%以上用于改善民生，财政民生支出累计达到2852.74亿元，是“十一五”的3.7倍。扎实办好每年“十项惠民实事”，大力推进“十项清零行动”，累计新增城镇就业62.95万人，完成农村劳动力转移就业443.69万人次，分别比“十一五”增加4.26万人和145.29万人次，城乡常住居民人均可支配收入分别达到33188元和14072元，是2010年的1.8和2.0倍；完成86个城中村、33个棚户区征收搬迁，回迁群众26万人；开工建设保障性安居工程57万套，完成农村危房改造3.6万户，解决了全市40.1万户中低收入家庭住房问题，受益群众达到130万人；52.7万贫困人口按现行标准实现脱贫，组织实施移民搬迁9.4万人；新建农村饮水安全工程1048处，在全省率先全面解决了规划内农村居民饮水安全问题。教育投入持续增长，累计达到522.6亿元，是“十一五”的3.1倍，新建、改扩建标准化幼儿园528所，建成义务教育标准化学校496所，浐灞一中和丝路学校2所民族学校建成投用，实施了36所开发区学校、幼儿园建设，营养改善计划惠及学生38.4万人。完成了市中医医院、市胸科医院等6所医院的新建、迁建工作，新农合参合率和城镇基本医疗保险参保率分别达到99.2%和98.5%，城乡居民大病保险全面推开。建立城乡统一的居民基本养老保险制度，参保率达到99.9%。新增养老机构63家、农村幸福院412个、城市社区养老服务设施447处，全市养老服务床位达到3.7万张。先后5次提高城乡低保及农村五保供养标准，出台改善城区困难群众生活“22条措施”，更多特殊群体的突出问题得到妥善解决。“平安西安”创建活动成效明显，建立完善了立体化社会治安防控体系，三环内68个警务站投入使用，全市电梯安全监控平台（96333）建成运行，国家食品安全城市创建工作扎实有效，全国质量强市示范城市通过验收，连续2届荣获全国社会管理综合治理最高奖项“长安杯”。获得全国“双拥模范城市”七连冠，民族、宗教、参事、人防、档案、地方志、防震减灾等领域工作都得到全面加强，连续四年荣获“中国最具幸福感城市”称号。

——政府建设得到新加强。深入开展党的群众路线教育实践活动和“三严三实”专题教育，严格执行中央八项规定和国务院“约法三章”，大力整治“文山会海”和庸懒散奢等不良风气，整改完成市政府班子“四风”问题13件、“不严不实”问题11件，清理议事协调机构228个，政府执行力和公信力不断提升。坚持市政府常务会学法制度，不断规范科学民主决策程序，全面落实行政执法责任制。自觉接受人大及其常委会的依法监督和政协的民主监督，5352件建议、提案全部按时办复。全面实行政务公开和“三公”经费预决算公开，切实加大行政监察和审计监督力度，严格落实“两个责任”，认真履行“一岗双责”，不断推进党风廉政建设和反腐败斗争，严查各类违纪违法案件和损害群众利益的行为。

“十二五”发展成就令人鼓舞，但前进道路上我们面临的困难和问题依然很多。主要是：经济总量依然偏小，增速持续放缓，投资增长出现拐点，产业转型升级任务艰巨，新旧动力转换尚需时日，实体经济的发展环境、要素保障需要不断优化提升。发展不平衡、不协调的矛盾还比较突出，城乡之间基础设施、公共服务、居民收入还有较大差距。城市治理水平需要进一步提高，交通拥堵、环境污染等问题仍需持续用力。在政府自身建设方面，不仅引领经济发展的能力需要持续提高，廉而不勤、严而慢为、不敢担当等问题更需要从严查纠。

各位代表，过去的五年，我们经受了前所未有的考验，也取得了令人瞩目的成就。这是省委、省政府和市委正确领导的结果，是市人大、市政协监督支持的结果，是全市人民团结奋斗的结果。在此，我谨代表市人民政府，向全市人民、驻西安部队指战员、武警官兵和公安民警致以崇高敬意！向大力支持政府工作的各位代表、各位委员和社会各界人士，向所有关心和支持西安发展的海内外朋友表示衷心感谢！

二、“十三五”发展的基本思路和目标任务

“十三五”是西安全面建成小康社会的决胜期和初步建成具有历史文化特色的国际化大都市的关键期。根据国家和省上有关“十三五”的总体安排和对西安发展的要求，市政府编制的《西安市国民经济和社会发展第十三个五年规划纲要（草案）》，提出了未来五年的指导思想、发展目标、主要任务和重大措施，本次大会审议批准后，我们将认真组织实施。

总的考虑是，在“十三五”时期，要深入贯彻创新、协调、绿色、开放、共享的发展理念，按照“追赶超越”和“五个扎实”的要求，系统推进品质西安建设，实现经济实力、城乡居民收入、经济外向度三大跃升，促进城市创新驱动力、产业竞争力、综合承载力、辐射带动力及绿色发展力五方面能力大幅增强，确保在全省率先全面建成小康社会，努力当好“三个陕西”建设的“排头兵”。

建设承古开新、开放包容、高端优质、和谐宜居的品质西安，是我市全面建成小康社会、全力打造丝绸之路经济带新起点和国际化大都市的战略选择，是深入调整结构、全面提高发展质量和效益的必然要求，也是顺应全市人民对美好生活的新期待、共同创造和谐宜居美丽家园的现实需要。要把品质西安建设，作为我市深入贯彻党的十八届五中全会和中央经济工作、城市工作会议精神，全面落实“五大发展理念”及“中国特色城市发展道路”的具体实践，贯穿于经济社会发展的各个领域和全面建成小康社会的全过程，着力提升经济发展、城市治理、宜居环境、对外开放、人民生活和政府服务等“六大品质”，真正让广大市民从品质建设中看到变化、得到实惠。

着力提升经济发展品质。必须牢牢把握发展是第一要务，聚焦转型升级和追赶超越，把创新摆在引领发展的核心位置，着力加强供给侧结构性改革，大力实施质量强市战略，以高端优质为导向，充分发挥历史、文化、科教、旅游等资源优势，基本建成具有西安特色的现代产业新体系。正确处理保持合理增速与提高质量效益的关系，做到调速不失速、量增质更优，确保经济增速稳居全国副省级城市“第一方阵”，加快迈向“GDP万亿俱乐部”。“十三五”，全市生产总值年均增长8%以上，人均生产总值超过1.5万美元，建成六大千亿级战略性新兴产业集群，服务业增加值占GDP比重达到60%以上，科技进步贡献率提高到63%。

着力提升城市治理品质。必须牢牢把握传承保护与创新发展相统一的原则，彰显特色风貌，推进“多规合一”，实现全域统筹，全面提升城市规划、建设、管理水平。转变城市发展方式，坚持系统思维，切实加强对城市工作的全方位、全过程、全要素治理，以智慧城市建设为重点，加快推进城市治理体系和治理能力现代化，有效解决各种“城市病”，确保城市安全，提高文明程度，着力打造具有历史延续性、地域识别性和“国际范”的现代化城市。到2020年，四级城镇体系更加完善，现有棚户区改造全面完成，城市精细化管理水平大幅提升，初步建成具有历史文化特色的国际化大都市。

着力提升宜居环境品质。必须牢牢把握生态文明的理念，着力提高城市发展的持续性和宜居性，推动形成绿色低碳的生产生活方式和城市建设运营模式，实现生产空间集约高效、生活空间宜居适度、生态空间山清水秀。按照“把好山好水好风光融入城市”的要求，持续抓好秦岭生态保护、“八水润西安”、城市生态廊道建设等重大生态治理工程，将风道建设纳入城市规划和管理，加快构建山、水、塬、田、城和谐共生的生态格局，坚决打好治污减霾持久战，努力为市民创造天更蓝、地更绿、水更清的宜居家园。到2020年，全市森林覆盖率达到48%以上，单位GDP能耗降低16%，空气质量优良率达到80%以上。

着力提升对外开放品质。必须牢牢把握开放兴市的基本方略，以打造“内陆型改革开放新高地”和“中国向西开放新窗口”为目标，主动融入“一带一路”大格局，全面落实“五通”举措，坚持优进优出，不断增创对外开放新优势，积极拓展“海外西安”发展新空间，着力构建全方位、高水平对外开放新格局。到2020年，全市进出口总值突破500亿美元，外贸依存度达到30%，开通国际航线达到50条，西安对外的知名度、美誉度和国际影响力大幅跃升。

着力提升人民生活品质。必须牢牢把握以人民为中心的发展思想，把增进百姓福祉作为发展的出发点和落脚点，扎实办好每年“十项惠民实事”，全面补齐贫困“短板”，各项社会保障制度更加科学完善，使城市更具包容性。增加基本公共产品和公共服务的有效供给，实现学有所教、劳有所业、病有所医、老有所养、住有所居，促进人的全面发展，让每个勤奋的人在西安都有机会、都能出彩、都可以成功，让每个怀揣梦想的人在西安都有筑梦空间、都会为梦奋斗、都能梦想成真。到2020年，累计城镇新增就业60万人，农村劳动力转移就业300万人次，城乡常住居民人均可支配收入分别达到48000元和21000元，全面实施13年免费教育，新增劳动力平均受教育年限达到14.9年以上，每千人医疗机构床位数和执业医师数分别达到7.49张和3.39人。

着力提升政府服务品质。必须牢牢把握为民、务实、清廉的要求，巩固深化党的群众路线教育实践活动和“三严三实”专题教育成果，以建设法治政府、创新政府、廉洁政府和服务型政府为抓手，持之以恒推进作风建设，不断强化责任担当，全面提升政府服务能力和水平，积极营造法治化、国际化、便利化的优质营商环境，全力维护社会公平正义，有效发挥市场在资源配置中的决定性作用，努力为西安追赶超越发展创造良好环境。到2020年基本建成职能科学、权责法定、执法严明、公开公正、廉洁高效、守法诚信的法治政府，公众满意率大幅提升。

三、2016年主要任务

今年是实施“十三五”规划的开局之年，也是我市系统推进全面创新改革试验的起步之年，我们将以品质西安建设为主线，以供给侧结构性改革为统领，突出抓好去产能、去库存、去杠杆、降成本、补短板五大任务，努力在全省追赶超越发展中起到示范、带动、辐射、支撑作用。

全市经济社会发展的主要预期目标是：生产总值增长8%以上；规模以上工业增加值增长7%左右；社会消费品零售总额增长10%左右；全社会固定资产投资增长5%左右；进出口总值增长8%左右；一般公共预算收入增长11%；城镇登记失业率控制在4.5%以内；城乡常住居民人均可支配收入分别增长8%和9.5%左右；CPI涨幅控制在3%左右；单位GDP能耗下降3%，主要污染物排放指标控制在规定范围以内。

（一）扎实推进全面创新改革，为追赶超越发展增加新动能。

全力推进两个“国字号”试验示范工作。重点抓好军工企业所有制改革、军工院所事转企改革、高校院所技术转移转化制度改革等试点，启动实施一批重大产业项目，做大做强军民融合高新技术产业集群。总结推广西安光机所、西北有色院科研、中试、产业“三位一体”经验，加强共性关键技术产学研协同创新，提升科研院所产业溢出效应。深化科技金融结合，在知识产权质押融资、投贷联动和股权众筹等领域组织先行先试。抓好西北技术转移中心和西部知识产权运营平台两个国家级平台建设，打造市场化、专业化和网络化的西安科技大市场，力争入网仪器设备突破1万台套，技术成果交易额超过700亿元。深入实施培育科技企业小巨人三年行动计划，打造100家小巨人领军企业。大力推进大众创业、万众创新，构建多层次创业创新空间，让全市热衷创新创造的创客和极客们大展身手，让更多有生命力的前沿技术和新兴产业集群蓬勃发展。统筹做好人才引进、培养和使用工作，推进“外专千人计划”和高端外国专家项目，加快西安人力资源服务产业园建设，完善创新人才评价和激励机制，健全政府配套服务体系，营造鼓励创新、崇尚创业、宽容失败的良好环境，增强对各类人才的吸引力。继续开展创业大赛和“西安双创活动周”等活动，厚植创业创新文化。

着力加强供给侧结构性改革。围绕降低制度性交易成本、提高供给体系质量和效率、提高投资有效性等重点，进一步放松管制、释放活力，让市场发挥更大作用。按照简政放权、放管结合、优化服务、转变政府职能的要求，推进行政权责依法公开，6月底前公布我市权力清单、责任清单、负面清单。引导企业顺应市场需求变化，加大技术改造更新力度，增加优质新型产品和生活服务等有效供给。放宽市场准入，大力推进PPP模式，鼓励民营资本进入更多领域。继续深化商事制度改革，抓好先照后证、“三证合一”“一照一码”等制度落实，放宽企业经营范围核准和住所登记条件，全市工商注册企业总量突破24万户。深入开展“千人扶千企”活动，着力解决企业运营中的实际困难，加大对本地绩优企业和各领域领军企业的政策扶持，确保全年净增规模以上工业企业40户、限额以上贸易企业80户、规模以上服务业企业60户，形成各类企业梯次跃进的竞相发展态势。着力打好降低企业成本“组合拳”，全面落实国家各项减免税政策，加快建立涉企收费清单，清理各种不合理收费，切实减轻企业负担，让企业有更多利润。继续完善政府、金融机构、担保机构和企业共同参与的“四方合作”机制，进一步拓宽企业融资渠道，新增贷款2200亿元。依法打击非法集资行为，切实保护投资者合法权益。完成市属国有企业分类工作，推进薪酬制度改革，加快市属企业上市及并购重组，提高国有资本证券化率。

深入推进重点领域改革。进一步厘清开发区与行政区的管理权责，推动开发区优化整合，启动部分行政区区划调整前期工作。不断完善城市管理体制，按照“规划上移、管理下沉”和“人随事走、费随事转”的原则，落实属地化管理责任。深入推进农村综合改革，全面完成土地确权登记颁证工作，启动市级农村产权流转交易平台建设。积极稳妥推进事业单位分类改革。继续推进财政预算改革，强化地方政府债务管理。健全社会信用体系，推动全过程信用管理。推进政府购买公共服务，加快供水、供气、供热等价格市场化改革步伐。

（二）全力以赴稳增长，确保经济运行在合理区间。

强力推进招商引资。把招商引资作为“一把手工程”，按照全市“一盘棋”的思路，积极从外引、向上争、朝内挖，明确任务，严格奖惩。坚持招大引强选优，有针对性地组织点对点招商，努力吸引更多产业链长、科技含量高、税收贡献大的好项目、大项目落户我市，全年实际利用外资42亿美元、引进内资1680亿元以上。紧跟国家产业政策和投资方向，围绕重大基础设施建设、产业转型升级、生态环境改善和社会事业发展等方面，从中、省争取一批重大项目和配套扶持资金，继续抓好央企进陕项目落实。积极向内挖潜，鼓励存量资本扩张发展，支持三星、比亚迪、陕重汽、西飞等龙头企业延伸产业链，策划包装一批含金量高、收益预期好的重大项目，带动相关配套企业加速聚集，不断夯实发展的后劲。积极营造亲商、扶商、安商的投资发展环境，重视发挥企业家作用，重奖一批对全市经济发展有突出贡献的优秀企业家，大力营造企业家功高、纳税者光荣的社会氛围。

着力扩大有效投资。全力抓好560个市级重点项目建设，确保完成投资1825亿元以上。进一步优化投资结构，确保产业项目占比达到50%以上。将市级财政支持产业发展资金，重点投向传统制造业改造升级、战略性新兴产业和现代服务业等新领域，以及渭北工业区、金融商务区和“众创空间”孵化基地等新板块。用好用足国家专项建设债券和西安合作发展基金，在城棚改、保障房等民生领域，以及地铁、市政公用设施等建设方面，推进一批PPP示范项目，年内完成城建投资419亿元。不断完善重点项目领导包抓、部门负责、督查跟进制度，严格落实立项审批、土地指标、征地拆迁等责任，有效解决项目落地难、阻力大、进度慢等问题。深入开展投资环境专项整治行动，坚决打击强揽工程、阻挠施工等违法行为。

加快释放消费新需求。积极培育健康、养老、休闲等新消费领域，鼓励发展租赁、网购、定制等新型消费业态和消费模式，促进消费升级。加快改造提升曲江、大兴、龙首等重点商圈，大力发展城市商业综合体，曲江芙蓉新天地、高新中大国际建成投用，重点打造古都印象、永兴坊（二期）、老城根等特色商业和美食文化街区。支持我市知名商贸、餐饮企业开展网上营销，推动电子商务与传统商贸企业融合发展。促进电商向农村发展，加快实施“快递下乡”工程，释放农村消费潜力。着力化解房地产库存，落实我市“楼市新9条”，以满足新市民需求为基点，建立购租并举的住房制度，重点做好来我市创业、就业人员的住房服务和政策支持，推动棚改安置房、公租房和租赁市场与存量商品房市场有效衔接，引导房地产企业不断改善产品结构，积极顺应市场规律调整营销策略，适当降低商品住房价格，进一步发挥公积金对合理住房消费的支持作用，促进房地产市场健康发展。

（三）紧盯高端优质，强力推动产业转型升级。

继续深入实施工业突破战略。全面落实我市贯彻“中国制造2025”《实施意见》，积极运用“互联网＋”理念，推动制造业向高端化、智能化迈进。实施渭北工业区各组团基础设施互联互通工程，加快三星二期、新舟700、北斗卫星导航、西安金龙汽车等重大项目建设，突出抓好146个投资过5000万元的重点工业项目，确保年内竣工投产30个以上，工业投资增长8%以上。大力发展战略性新兴产业，在继续做大做强高端装备、航空航天等优势产业的同时，围绕节能与新能源汽车、智能终端、半导体等产业领域，实施“新千亿级产业集群培育计划”，建设世界一流高端芯片制造、封装测试一体化产业基地，力争战略性新兴产业增加值增长15%左右。

加快旅游业转型升级。围绕创建中华古都文化国际旅游目的地这个目标，持续加大资金投入，积极整合各类旅游资源，从交通规划、城市设计、景观打造、服务设施、旅游产品等多方面着手，促进全域旅游发展，不断提升西安旅游的内涵和品质。着力完善“智慧旅游”体系，启动西安旅游（云）数据中心建设，健全旅游智能服务系统。大力发展生态旅游、民俗体验、红色旅游和研学旅游等新型旅游业态，支持旅游商品创意研发和产业化发展。积极实施“旅游精品工程”，狠抓景区提档升级，加快城墙·碑林历史文化景区创建5A级景区步伐。推动旅游与文化融合发展，深入挖掘西安历史、民俗、宗教和山水文化等资源，重点抓好汉长安城文化景区、白鹿原文化旅游区、航空旅游度假区等项目建设，联合丝路沿线城市打造一批高品质文化旅游产品，构建以西安为起点的丝绸之路风情体验旅游走廊。进一步优化旅游环境，重拳整治黑车、黑导、黑社等旅游市场乱象，力争年接待海内外游客1.5亿人次，实现旅游总收入1200亿元。

推动服务业创新发展。继续抓好8个国家级和24个省市级服务业试点聚集区建设，促进生活性服务业向精细化和高品质转变，推动生产性服务业向专业化和价值链高端延伸，全年服务业增加值增长9%左右。启动建设丝路国际会展中心，促进会展业与其他新型业态联动发展，着力打造具有世界影响力的会展名城，全年举办规模以上会展活动180个。加快新筑铁路综合物流中心、西北金属物流工业园等现代物流聚集区建设，开展城市共同配送试点，推进多式联运，打造区域物流中心。推动西安金融商务区和高新科技金融、曲江文化金融等产业聚集区加快发展，积极引进各类金融机构，促进互联网金融健康发展，完成西安民间金融街二期建设，年内挂牌上市企业20家，力争金融业增加值增长10%以上。以国家现代服务业综合试点为抓手，加快推进科技服务业集群发展，全年科技服务业增加值突破430亿元。深化文化科技融合，重点抓好智慧动漫文化产业园、陕西大剧院等项目建设，推出一批代表西安文化特色的大剧大片和精品力作，支持以关中民俗博物院为代表的民营文化企业发展，全力办好第十一届中国艺术节，文化产业增加值增长9%以上。

大力发展都市型现代农业。出台我市《加快转变农业发展方式实施意见》，推动农村一、二、三产业交叉融合。加快推进“一区三带七板块”建设，发展大田托管、农产品加工、仓储物流等市场化服务，年内认定扶持市级以上现代农业园区15个，新增市级以上龙头企业10家、家庭农场80个。深入实施农业提质增效“五大工程”，建设粮食高产万亩示范片40个、千亩示范方100个、畜禽粪污资源化利用示范工程40个，全年休闲农业吸纳旅游人数达到1500万人次以上，经营收入超过18亿元。支持龙头企业带动农民发展多种形式的适度规模经营，采取“保底＋分红”的方式，让农户分享加工销售环节收益，力争全市农民人均可支配收入突破15000元。健全农产品质量安全监管和追溯体系，农产品质量安全抽检合格率稳定在96%以上。

（四）全面加快“新起点”建设，构建高水平对外开放新格局。

完善提升载体功能。落实我市“一带一路”建设《行动计划》，以欧亚经济论坛综合园区核心区建设为重点，加快推进论坛发展实体化进程。持续增强西安港功能，进一步完善口岸监管、检验等设施，年内建成粮食和肉类口岸，争取整车进口口岸获批。加快发展跨境电子商务，建设丝绸之路经济带西安国际采购中心。支持国际港务区建设融资租赁业聚集区，率先进行融资租赁业政策、功能和制度创新。以增量延线为重点，着力提高“长安号”运行质量。积极配合省上做好自贸区申报工作。

扩大区域务实合作。落实《西安宣言》确定的合作事项，加快欧亚创意设计园等115个重点项目建设。协力筹办第20届西洽会暨丝博会，继续办好丝绸之路经济带城市圆桌会，打造国际合作发展实体平台。力争开通墨尔本、旧金山、迪拜等国际航线，推动空港陆港联动发展。支持市内企业、高校和民间组织广泛开展对外交流活动，办好第三届丝绸之路国际电影节。

深入实施“走出去”战略。引导支持有实力的本地企业开展跨境业务，推动我市机械装备、汽车制造、电子信息等优势产业走出国门，鼓励外贸企业在丝路沿线国家建立营销、服务网络，积极拓展海外市场。进一步优化出口商品结构，扩大高技术、高附加值、高效益产品出口，加大对超导材料、输变电设备和工业缝纫机等高新技术和自主品牌的扶持力度，提升出口产品核心竞争力。

积极增创对外开放新优势。整合优化海关特殊监管区域，申建陕西航空综合保税区。大力发展对外加工、服务外包等外向型产业，打造国际服务外包名城。加快建设国际学校、医院和社区，不断完善城市涉外综合服务功能，提高服务水平。推进西安领事馆区建设，确保10月底前首批4座统建馆建成投用，力争马来西亚驻西安总领馆年内开馆。

（五）坚持以人为核心，加快推进新型城镇化。

进一步优化城市发展格局。树立“精明增长”“紧凑城市”理念，制定《西安市新型城镇化规划》，科学划定城市开发边界。切实做好与西咸新区的规划统筹，促进区域互联互通、协同发展。进一步强化城市规划管理，维护规划的严肃性和权威性，实现控制性详规、约束性体系规划全覆盖。以“城中河”的标准规划建设渭河，严格控制两岸生态红线和建筑、景观设计，年内启动渭河西安段滩区整治，确保南岸124公里堤顶路5月底全线通车。适时开展渭北工业区规划修改工作，坚持完善城市功能与发展重点产业并重，加快渭北新城建设步伐。推进主城区优化提升、内涵式发展，精心打造“十大城市片区”，着力提高服务功能和承载能力。加快推进“三个副中心”和“五个组团”建设，支持高陵、阎良开展新型城镇化改革试点，继续加强9个重点示范镇和6个文化旅游名镇建设，逐步形成横向错位发展、纵向分工协作的城镇发展格局。

大力彰显城市特色。加强城市空间立体性、平面协调性、风貌整体性、文脉延续性等方面的规划和管控，做好城市设计，促进建筑物、街道立面、天际线、色彩和环境更加协调、优美，留住城市特有的文化“基因”，坚决杜绝“洋、怪、丑”建筑。持续加大对古城墙、各类遗址和文物的保护力度，切实抓好大唐东市、丰镐、杨官寨、栎阳城等重点遗址的保护利用工作，推进汉长安城大遗址保护特区建设。深入实施古城复兴计划，加快北院门、三学街、七贤庄等历史文化街区改造提升。加强非物质文化遗产保护，全面开展优秀近现代建筑摸底建档工作。

不断完善城市功能。实施路、管、线“三网”畅通工程，着力突破“断头路”“卡脖子”等“瓶颈”，促进城市基础设施互联互通。继续强力推进缓堵保畅工作，年内启动第三轮地铁建设规划编制工作，3号线通车试运营，6、9号线开工建设，4、5号线和1号线延伸段加快推进。完成学府环路、朱宏路—凤城四路立交等一批改续建工程，开工建设西阎快速通道和8条市政道路，打通开元路、凤城七路等5条断头路。进一步优化公交线网，加快非机动车道建设，新建出行车位5万个，新增、更新公交车1100辆、投放公共自行车1万辆，适时启动城南客运站二期工程。协力推动西安火车站改扩建及西成高铁、西阎城际铁路等项目建设。加快推进城市地下综合管廊和海绵城市建设，完成中心城区地下管线普查工作和丈八北路等20项架空线缆落地工程，实施16项排水管网及积水点改造项目。完成李家河水源工程收尾、大峪引水提升等一批重点水利工程，启动建设西南郊水厂。

推进城市治理现代化。以“智慧城市”建设为抓手，加强信息技术应用和各类政府信息资源整合，重点实施智慧交通、智慧城管、智慧社区建设，构建西安“大数据”服务平台，逐步实现城区公共场所Wifi全覆盖。深入推进城市精细化管理，扎实做好“国家卫生城市”复检，大力开展市容环境集中整治行动，着力解决一批群众反映强烈的城市管理顽疾，创建“百条整洁美丽示范街”。配套推进户籍制度改革，出台我市居住证管理办法，实行积分落户制度。加强流动人口和特殊人群服务管理。开展物业市场专项整治。依法组织好社区居委会换届工作。鼓励企业、市民和社会组织通过各种方式参与城市建设、管理，真正实现城市共治共管、共建共享。

（六）突出生态宜居，开展“美丽西安·绿色家园”行动。

持续用力推进治污减霾。年内新建、扩建8座污水处理厂，新增日污水处理能力71.5万吨，再生水利用率达到18%，实现渭河西安段水质稳定达标。推动治污减霾由“治标”向“治本”转变，不断巩固“清零”成果，加大新能源推广力度，年内削减燃煤总量40万吨，淘汰黄标车1.9万辆，做好重污染天气应急响应。实施工业污染深度治理和重金属污染防治，推动企业环保设施改造升级，开展臭氧超标专项整治，依法严惩各类环境违法行为。全年二级以上优良天数不少于251天。

深入实施生态优化提升工程。积极创建全国水生态文明试点城市，加快推进“八水润西安”工程，着力抓好新一轮渭河综合治理，继续加大浐河、泾河、清河等河流治理力度，启动涝河渼陂湖生态修复，新增生态水面2450亩、湿地6600亩。依法加强秦岭生态保护，重点抓好源头治理和主要峪口、新环山路、河道景观提升改造，加快植被恢复和直面陡坡绿化，打造秦岭生态金字招牌。实施园林绿化景观提升工程，加快幸福林带等城市生态廊道建设，改造提升兴庆宫、莲湖、纺织等6座老公园，启动建设氵皂河生态公园、杜陵生态遗址公园等一批新公园，加快曲江文化运动公园、潏河湿地公园建设，西安植物园新址（一期）和雁鸣湖公园建成开放，新建、提升街头绿地广场100个，新增城市绿地400万平方米、造林3万亩，确保创建国家森林城市通过验收。

建立生态保护长效机制。严格划定生态保护红线，探索建立周至、蓝田重点生态区生态补偿机制，制定出台《森林资源保护管理责任制办法》。建立生态文明综合评价指标体系，实行领导干部自然资源资产离任审计。大力发展绿色产业、绿色企业和循环经济，推广绿色建筑和建材，推进各类园区循环化改造和再生资源回收体系建设。做好餐厨废弃物资源化利用和无害化处理全国试点，提高建筑垃圾综合利用能力，生活垃圾无害化处理率城市保持100%，县城达到85%。

巩固提升美丽乡村建设成果。以创建“西安最美乡村”活动为抓手，大力开展农村环境综合整治工作，重点抓好村庄保洁和垃圾、污水无害化处理。启动幸福新农村示范村建设工作，开工建设100个农村片区化中心社区。年内创建市级以上生态镇村50个。

（七）办好惠民实事，努力让全市人民有更多获得感。

继续抓好扶贫开发工作。巩固全面脱贫成果，在更高起点上推进精准扶贫和精准脱贫。按照“五个一批”和“六个精准”的要求，突出抓好218个低收入村和9916户低收入户的精准帮扶，启动2377户、8821人的移民搬迁工作。狠抓产业扶贫，实施电商、旅游等扶贫工程，打造20个旅游扶贫示范村。以“美居行动”为抓手，着力解决城区困难群众居住、就业、看病、上学等急难问题，全面开展城市扶贫工作。

扎实办好人民满意教育。继续深化教育领域综合改革，促进基础教育均衡发展。完成第二期学前教育三年行动计划任务，年内新建、改扩建幼儿园102所。完成大学区优质资源共享平台三期和中小学在线课堂建设，实现“校校通”全覆盖。大力发展职业教育、特殊教育、民族教育和继续教育，促进各级各类教育全面协调发展。

加快健康西安建设。制定实施《健康西安行动计划》，全面实行分级诊疗制度，加强以全科医生为重点的基层医疗卫生队伍建设，推动医疗资源下沉。启动城市公立医院综合改革试点，深化医疗联合体建设，推动互联网医疗产业发展，鼓励社会办医，健全重特大疾病救助和疾病应急救助制度，下大气力解决群众看病难、看病贵问题。落实全面两孩政策，改革完善计划生育服务管理。

筑牢民生保障安全网。坚持就业优先，以创业带动就业，重点抓好高校毕业生、农村富余劳动力、城镇失业人员等重点群体就业，积极实施“四大创业计划”和农民工返乡创业“7项行动计划”，全年城镇新增就业11万人，农村劳动力转移就业65万人次。加大城镇棚户区、老旧住宅小区和农村危房改造力度，进一步完善保障房基础配套，年内提供保障性住房房源2万套，改造老旧小区100万平方米、农村危房3500户。落实我市“棚改新8条”，不断扩大货币化安置比例，启动一批棚户区改造项目，完成3.05万群众回迁安置。建立社会保险待遇水平联动增长机制。整合城乡居民基本医疗保险制度。继续推进机关事业单位养老保险制度改革。实施全民参保登记计划，确保城镇基本医疗保险参保率保持在96%以上，城乡居民养老保险参保率保持在99%以上。适时提高城乡低保等各类保障标准，完善社会救助制度，让困难群众求助有门、受助及时。

系统推进社会事业发展。加快构建以居家为基础、社区为依托、机构为支撑的养老服务体系，全面推进医养结合，建成城市社区居家养老服务站122个、农村社区养老服务站189个。加快西安图书馆新馆、广播电视发射中心建设，组织戏剧惠民演出1100场。启动市体育场改造项目，抓紧筹建西安奥体中心，加快建设区县标准足球场，新建全民健身路径150个。畅通群众利益协调、权益保障法律渠道，创建300个示范化社区（村）公共法律服务室。深入开展双拥创建活动，继续抓好民族、宗教、人防、地方志、档案等工作。

全力维护城市安全。深入推进“平安西安”建设，依法严厉打击暴恐、黄赌毒、涉众型经济犯罪等违法犯罪活动，提高公众安全感。落实安全生产“党政同责、一岗双责、失职追责”，建立隐患排查治理动态监管体系，切实加强对地铁、高层建筑、特种设备、市政公用管线、危险化学品和人员密集场所等的安全监管，扎实做好各类大型活动的安全风险评估与管控，坚决遏制重特大安全事故发生。继续抓好国家食品安全城市创建工作，切实加强对学校、医院周边和城中村等重点区域小作坊、小摊贩、小餐饮的综合治理，坚持疏堵结合，引导规范经营，努力让广大市民吃得安全、吃得放心。继续做好水源保护区划定工作，强化水质管控和应急能力建设，确保饮用水水源水质合格率达到优良以上。完善城市应急管理体系，提高气象、地质、地震灾害防御能力，筑牢城市安全“防火墙”。

（八）践行“三严三实”，全面建设人民满意政府。

坚持依法行政、从严治政。认真贯彻落实《法治政府建设实施纲要（2015—2020年）》，严格按照法定程序、法定权限行使职权，将政府各项工作纳入法治轨道。加强重点领域政府立法，深化行政执法体制改革，自觉接受人大依法监督、政协民主监督和社会舆论监督。落实公众参与、专家论证、风险评估、合法性审查和集体讨论决定相结合的重大行政决策机制，确保各项重大行政决策合法合规、符合群众意愿。加强各级行政决策、行政行为的责任倒查，对严重失误或者造成重大损失、恶劣影响的，依法追究责任。

坚持为民服务、高质高效。全面推行政务公开，积极开展“互联网＋政府服务”，推进全程网上审批，让群众少跑路、不跑路。继续办好“行风热线”、政府网站和领导信箱，倾听群众呼声和社情民意，开展政风行风民主评议，加强与人民群众的沟通联系。推行市民电视问政，对为官不为、懒政怠政的要公开曝光、坚决追责。形成上下贯通、层层负责的主体责任链，健全能定责、可追责的考核机制，条条线都要拉直绷紧。

坚持真抓实干、敢于担当。喊破嗓子不如甩开膀子。要始终保持奉公为民的热情、干事创业的激情和拼搏争先的豪情，为西安发展担当，为全市人民尽责，坚持低调务实不张扬、埋头苦干，不图虚名、不务虚功，多做打基础、利长远的事情，以“钉钉子”的精神推动各项工作落实。要探索建立合理的容错机制，把干部在推进改革中缺乏经验、先行先试出现的失误和错误，同明知故犯的违纪违法行为区分开来；把上级尚无明确限制的探索性试验中的失误和错误，同上级明令禁止后依然我行我素的违纪违法行为区别开来；把为推动发展的无意过失，同为谋取私利的违纪违法行为区分开来，保护那些作风正派又敢作为、锐意进取的干部，为担当者担当。

坚持清正廉洁、廉而有为。自觉向中央看齐，严格对照中国共产党《廉洁自律准则》和《纪律处分条例》，认真贯彻中央八项规定，落实“两个责任”和“一岗双责”，严控“三公”经费支出，持续深入反对“四风”。进一步健全惩治和预防腐败体系，加大财政资金分配、重大项目建设、重大物资采购招标等重点领域审计力度，实现审计监督全覆盖。始终保持反腐高压态势，对腐败分子和腐败行为零容忍、严查处，努力营造风清气正的政治生态。

各位代表！西安未来五年的发展蓝图已经绘就，追赶超越发展的号角已经吹响。让我们紧密团结在以习近平同志为总书记的党中央周围，在市委的坚强领导下，以更严更实的精神和作风，加快推进品质西安建设，为全面建成小康社会、加快建设具有历史文化特色的国际化大都市而努力奋斗！
